# Correction: N/O co-enriched graphene hydrogels as high-performance electrodes for aqueous symmetric supercapacitors

**DOI:** 10.1039/d1ra90124a

**Published:** 2021-06-22

**Authors:** Yong Zhang, Liang Wei, Xijun Liu, Wenhui Ma, Jiankai Wang, Shan Fan

**Affiliations:** College of Materials Science and Engineering, Graphene Functional Materials Research Laboratory, Qiqihar University Qiqihar 161006 P. R. China leon1981@163.com 15804528735@163.com; School of Chemistry and Chemical Engineering, Qiqihar University Qiqihar 161006 P. R. China; College of Materials Science and Engineering, Heilongjiang Province Key Laboratory of Polymeric Composition Material, Qiqihar University Qiqihar 161006 PR China

## Abstract

Correction for ‘N/O co-enriched graphene hydrogels as high-performance electrodes for aqueous symmetric supercapacitors’ by Yong Zhang *et al.*, *RSC Adv.*, 2021, **11**, 19737–19746, DOI: 10.1039/D1RA01863A.

The authors regret that an incorrect version of Scheme 1 was shown in the original article. The corrected version of Scheme 1 is shown below.

**Scheme 1:**
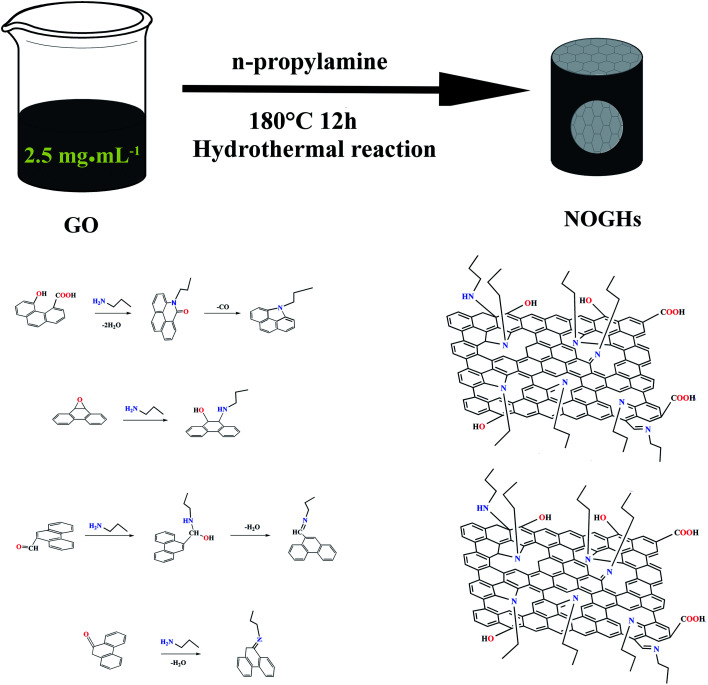
Illustration of the possible reaction mechanism of the NOGHs.

The Royal Society of Chemistry apologises for these errors and any consequent inconvenience to authors and readers.

## Supplementary Material

